# Metagenomic Analysis of Infectious F-Specific RNA Bacteriophage Strains in Wastewater Treatment and Disinfection Processes

**DOI:** 10.3390/pathogens8040217

**Published:** 2019-11-03

**Authors:** Suntae Lee, Mamoru Suwa, Hiroyuki Shigemura

**Affiliations:** Innovative Materials and Resources Research Center, Public Works Research Institute, Ibaraki 305-8516, Japan; suwa@pwri.go.jp (M.S.); h-shigemura@pwri.go.jp (H.S.)

**Keywords:** F-specific RNA bacteriophage strain, viral indicator, next-generation sequencing, infectivity, wastewater treatment, chlorination, ultraviolet disinfection

## Abstract

F-specific RNA bacteriophages (FRNAPHs) can be used to indicate water contamination and the fate of viruses in wastewater treatment plants (WWTPs). However, the occurrence of FRNAPH strains in WWTPs is relatively unknown, whereas FRNAPH genotypes (GI–GIV) are well documented. This study investigated the diversity of infectious FRNAPH strains in wastewater treatment and disinfection processes using cell culture combined with next-generation sequencing (integrated culture–NGS (IC–NGS)). A total of 32 infectious strains belonging to FRNAPH GI (nine strains), GI-JS (two strains), GII (nine strains), GIII (seven strains), and GIV (five strains) were detected in wastewater samples. The strains of FRNAPH GI and GII exhibited greater resistance to wastewater treatment than those of GIII. The IC–NGS results in the disinfected samples successfully reflected the infectivity of FRNAPHs by evaluating the relationship between IC–NGS results and the integrated culture–reverse-transcription polymerase chain reaction combined with the most probable number assay, which can detect infectious FRNAPH genotypes. The diversity of infectious FRNAPH strains in the disinfected samples indicates that certain strains are more resistant to chlorine (DL52, GI-JS; T72, GII) and ultraviolet (T72, GII) disinfection. It is possible that investigating these disinfectant-resistant strains could reveal effective mechanisms of viral disinfection.

## 1. Introduction

F-specific RNA bacteriophages (FRNAPHs), which are known to infect *Escherichia coli* that express F pili, have a single-stranded RNA genome enclosed in an icosahedral capsid measuring 20–30 nm in diameter. The sizes, shape structures, and genomes of FRNAPHs are similar to those of noroviruses [1,2], which have caused numerous outbreaks of gastroenteritis in multiple countries [3]. Furthermore, FRNAPH behavior, abundance, and survival in the environment including during water treatment are also similar to those of human enteric viruses [1,2,4,5,6]. Thus, they serve as potential indicators of water contamination and the fates of viruses in aquatic environments and wastewater treatment plants (WWTPs) [4,5,6].

FRNAPHs belong to the family *Leviviridae* and are classified into the genera *Levivirus* and *Allolevivirus*, which are subdivided into genotypes I and II (GI and GII) and genotypes III and IV (GIII and GIV), respectively. Each FRNAPH genotype has a different fate in WWTPs [6,7,8,9,10] and a different resistance to disinfection [11,12,13]. For example, genotypes GII and GIII are more prevalent than GI and GIV in municipal raw wastewater samples [6,8,9]. However, GI is the dominant genotype in the secondary effluent of WWTPs because of its higher resistance to wastewater treatment relative to other FRNAPH genotypes [6,8,9]. GI also showed the highest chlorine and ultraviolet resistance among the FRNAPH genotypes [11,12,13]. Particularly, MS2, belonging to GI, showed higher ultraviolet resistance than human pathogenic viruses (poliovirus, rotavirus, hepatitis A virus, and coxsackievirus) [13].

The presence and removal of FRNAPH genotypes in WWTPs have been the subject of numerous studies [6,7,8,9,10]. Moreover, several FRNAPH strains are included in each FRNAPH genotype and have been reported worldwide in bacterial isolates associated with sewage and mammal feces [14,15,16,17,18]. The sources from where different FRNAPH strains were first isolated/detected are shown in Table 1. In the last decade, novel GI-JS strains DL52 and DL54 were isolated, which are recombinant strains of environmental isolates of *Leviviridae* ssRNA bacteriophages [16]. Unfortunately, information regarding the occurrence of FRNAPH strains in WWTPs is relatively limited [19]. 

Numerous studies have employed MS2, GA, Qβ, and SP as representative FRNAPH strains of genotypes GI–GIV in spiking experiments to determine their surface properties, including electrostatic surface charge, hydrophobicity, and removal during water treatment processes such as coagulation and membrane filtration [20,21,22]. However, the dominance of these strains among the strains of each FRNAPH genotype is debated. Thus, it is particularly important to identify the dominant strains affecting the concentrations of FRNAPH genotypes.

FRNAPH GI and GIV predominantly occur in the feces and waste generated by animal farms, whereas FRNAPH GII and GIII are dominant in human feces and the raw sewage of WWTPs [23,24]. Thus, the distribution of FRNAPH genotypes has been widely studied in order to determine the source of fecal contamination in river water [7,25,26,27], shellfish [26,28,29], and sediments [27]. However, a previous study [19] suggested that this distribution is not sufficient for tracking the source of fecal pollution. The large diversity of FRNAPH strains in each genotype may be the reason for this limitation because they are found in a diverse range of water bodies (e.g., sewage, river water, and seawater), shellfish (oysters, mussels, and clams), and the feces of birds and mammals (including humans, chicken, swine, calves, and apes) [14,15,16,17,18]. For example, FRNAPH GI strains MS2, DL1, and J20 have been isolated from wastewater, river water, and chicken litter, respectively (Table 1). Therefore, it is important to investigate the diversity of FRNAPH strains.

Next-generation sequencing (NGS) is used to study viral metagenomes in different stages of wastewater treatment [30,31,32,33]. This method provides more conservative estimates of viral occurrence compared with the rates detected using quantitative polymerase chain reaction (qPCR) [32]. The advantage of metagenomics is that it allows a comprehensive characterization of FRNAPH strain diversity. However, like qPCR assays, metagenomic methods do not assess infectivity. Therefore, when samples acquired after disinfection using chlorine or ultraviolet light are subjected to NGS, the viral sequences do not reflect infectivity. Conversely, culture combined with PCR (integrated culture–PCR (IC–PCR)) can detect infectious viruses. For example, IC–RT-PCR combined with a most probable number (MPN) assay (IC–RT-PCR–MPN) has been used to quantitatively detect infectious FRNAPH genotypes [6,34,35]. Thus, we hypothesized that the application of NGS for detecting of FRNAPH strains propagated in a liquid medium may be effective for detecting infectious FRNAPH strains. NGS analyses of wastewater samples often show that the majority of genes are from eukaryotes and bacteria, which are more abundant than viruses and bacteriophages. However, propagating infectious FRNAPH strains in samples can result in large yields of FRNAPH sequences; it also differentiates between infective and inactive FRNAPH strains. Recently, known and novel plant viruses, which infect plants such as yams, were detected by NGS combined with robust yam propagation by tissue-culture [36]. NGS combined with cell culture was also used to characterize enteric viruses isolated from wastewater [33]. Thus, integrated culture–NGS (IC–NGS) can be used to detect infectious FRNAPH strains and high fractions of FRNAPH genes in wastewater samples.

To the best of our knowledge, this study is the first to use IC–NGS to investigate the diversity of infectious FRNAPH strains in wastewater treatment and disinfection processes. We prepared the influent and secondary effluent of a WWTP as well as disinfected secondary effluent (raw water) treated using chlorine or ultraviolet light. IC–NGS and IC–RT-PCR–MPN were performed to determine the diversity of infectious FRNAPH strains and the concentrations of infectious FRNAPH genotypes, respectively. The relationship between the results of the two assays was investigated to evaluate whether IC–NGS data can effectively reflect the infectivity of FRNAPHs.

## 2. Results

### 2.1. Metagenomic and Taxonomic Analyses

A summary of the metagenomic (BLASTn) and taxonomic (MEGAN) analyses is shown in Table 2. The numbers of reads of the 12 samples analyzed using IC–NGS ranged from 887,593 to 5,035,503, and the trimmed sequences were assembled into 611–18,941 contigs. The FRNAPH strains were represented in the contigs of all samples using IC–NGS, and 66–551 sequences represented the reference genomes of FRNAPH strains determined using BLASTn. The percentages of hits for FRNAPH strains relative to the number of contigs in the samples ranged from 3% to 36%. The vast majority of the hit sequences assigned using MEGAN represented bacterial sequences and ranged from 44% to 83%. Specifically, *Salmonella enterica* sequences dominated in the bacterial sequences (65–92% without 1127 influent sample). The range of contigs that did not correspond to a reference genome was 4–36%.

### 2.2. Detection of Infectious FRNAPH Strains in Wastewater Treatment and Disinfection Processes

**Figure 1:**
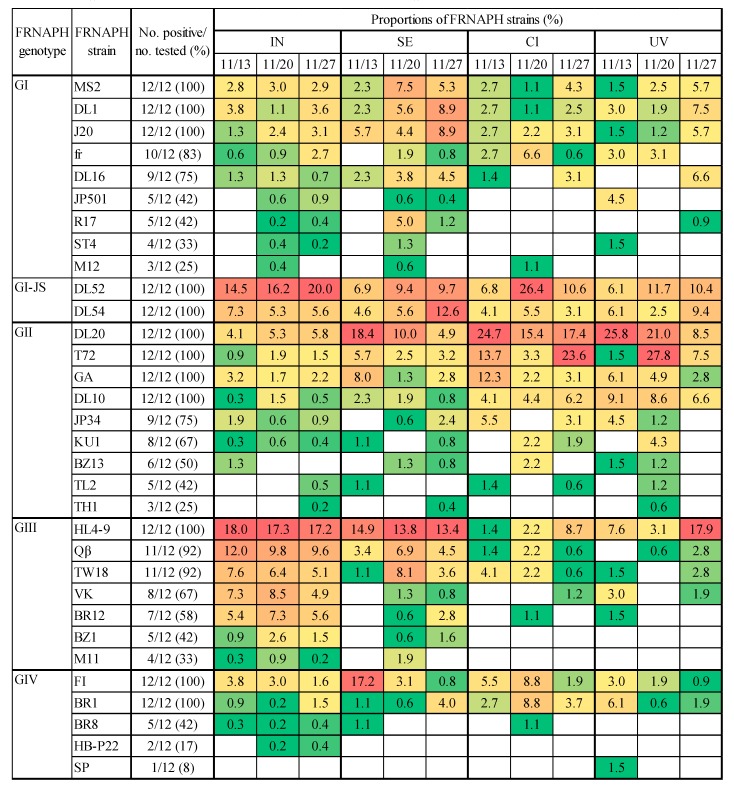
Proportions of FRNAPH strains representing each genotype in 12 samples combined with a heat map showing the relative abundance of all FRNAPH strains according to the number of hits (Supplementary Figure S1) in the BLASTn analyses of influent (IN), secondary effluent (SE), chlorine-treated (Cl), and ultraviolet-treated (UV) samples. Proportions (%) for FRNAPH strains were calculated as the number of hits for a specific FRNAPH strain relative to the total hits for all FRNAPH strains in each sample. Blank cells indicate an absence of hits. Green and red cells indicate the lowest and highest values, respectively. Numbers in the heat-map cells indicate the proportions for samples collected on 11/13, 11/20, and 11/27.

IC–NGS detected 31 stains representing all FRNAPH genotypes in influent, secondary effluent, chlorine-treated, and ultraviolet-treated samples on 11/13, 11/20, and 11/27 (Figure 1). The GI strains MS2, DL1, J20, fr, DL16, JP501, R17, ST4, and M12 were detected in all 12 samples (Figure 1). Specifically, MS2, DL1, and J20 were the most frequently detected GI strains (12/12, 100%). The proportions of GI strains in the secondary effluent samples were higher than those in the influent samples. The proportions of abundant GI strains (MS2, DL1, and J20) decreased from secondary effluent samples to chlorine- and ultraviolet-treated samples.

DL52 and DL54 (FRNAPH GI-JS) were detected in all samples (Figure 1). DL52 was the predominant strain of FRNAPHs in the influent samples together with HL4-9 (FRNAPH GIII). The proportions of DL52 decreased to a greater extent from influent to secondary effluent samples than those of DL54. In contrast, the proportions of DL54 decreased compared to those of DL52 from secondary effluent samples to chlorine-treated and ultraviolet-treated samples. The proportions of DL52 in chlorine-treated and ultraviolet-treated samples were similar or higher than those in the secondary effluent samples; specifically, the proportion of DL52 in the chlorine-treated sample from 11/20 (26.4%) was the highest among all FRNAPH strains.

The FRNAPH GII strains DL20, T72, GA, DL10, JP34, KU1, BZ13, TL2, and TH1 were detected in all 12 samples (Figure 1). Moreover, DL20 was the most predominant strain of FRNAPH GII in influent and secondary effluent samples (34.2–48.5% and 30.0–57.1% of the proportions in GII genotypes, respectively, Supplementary Figure S2). The proportions of GII strains in secondary effluent samples were higher than those in influent samples. DL20 had the highest proportion of all strains in the secondary effluent sample from 11/13 (18.4%). Furthermore, the proportions of FRNAPH GII strains in the chlorine-treated and ultraviolet-treated samples were similar or higher than those in the secondary effluent samples. Specifically, DL20 and T72 had the highest proportion in chlorine-treated and ultraviolet-treated samples from 11/13 (24.7% and 25.8%, respectively) and in chlorine-treated samples from 11/27 (23.6%) and ultraviolet-treated samples from 11/20 (27.8%), respectively.

The FRNAPH GIII strains HL4-9, Qβ, TW18, VK, BR12, BZ1, and M11 were detected in all 12 samples (Figure 1). HL4-9, which was detected in all samples (12/12, 100%), was the most abundant strain of FRNAPH GIII in all samples except chlorine-treated samples from 11/13 (28.6–83.3% of GIII genotypes, Supplementary Figure S2). Moreover, all FRNAPH strains in the influent samples together with DL52 represent FRNAPH GI-JS. The proportions of all strains of FRNAPH GIII in the influent samples was reduced by wastewater treatment (secondary effluent samples) and by chlorine (chlorine-treated samples) and ultraviolet disinfection (ultraviolet-treated samples), with the exception of ultraviolet-treated samples collected on 11/27 for HL4-9.

The FRNAPH GIV strains FI, BR1, BR8, HB-P22, and SP were detected in all 12 samples (Figure 1). FI and BR1 were the predominant FRNAPH GIV strains in all samples (Supplementary Figure S2). SP was detected only once in the ultraviolet-treated samples from 11/13. The proportion of FI increased to a greater extent from influent to secondary effluent samples on 11/13 compared to those on other dates, which were either similar or smaller. There were fewer hits for GIV strains in chlorine-treated and ultraviolet-treated samples (<9, Supplementary Figure S1).

### 2.3. Comparison of IC–RT-PCR–MPN and IC–NGS Data

**Figure 2:**
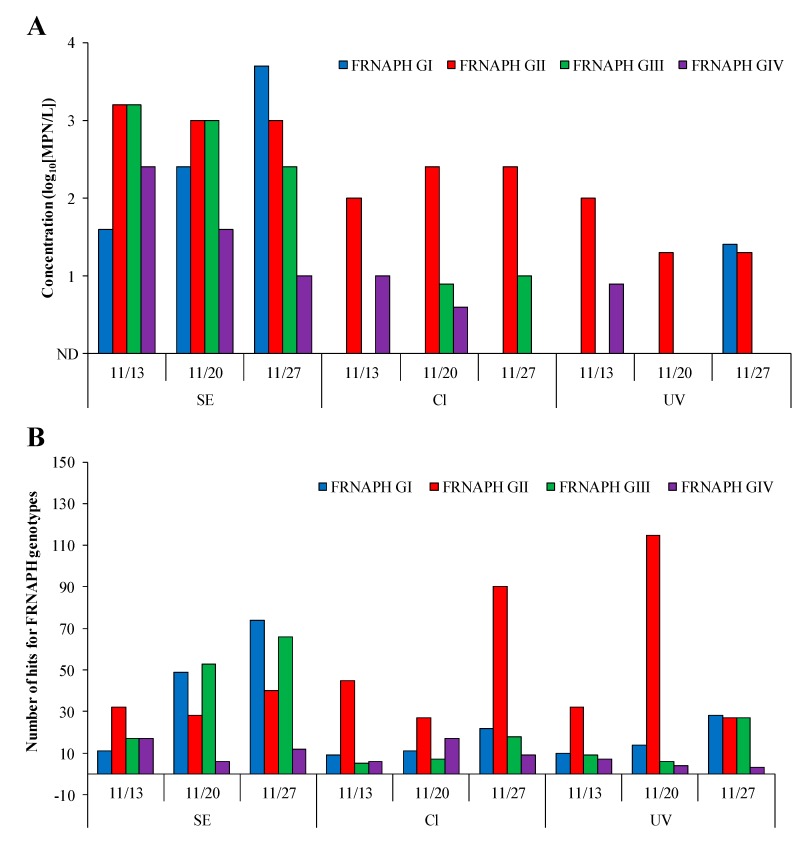
Concentrations of infectious FRNAPH genotypes determined using IC–RT-PCR–MPN (**A**) and number of hits for each FRNAPH genotype determined using IC–NGS (**B**) in the secondary effluent (SE), chlorine-treated (Cl), and ultraviolet-treated (UV) samples collected on 11/13, 11/20, and 11/27. Numbers of hits for each FRNAPH genotype represent the sum of the number of hits for FRNAPH strains of each genotype except GI-JS.

The relationship between the results for infectious FRNAPH genotypes detected using IC–RT-PCR–MPN and IC–NGS was investigated to determine whether IC–NGS effectively reflects the infectivity of FRNAPHs. In the IC–RT-PCR–MPN results (Figure 2A), infectious FRNAPH GII was detected in all chlorine-treated samples, whereas GI was not detected. GIII and GIV were detected in chlorine-treated samples collected on 11/20 and 11/27 and 11/13 and 11/20, respectively. GI and GIII were inactivated more effectively by chlorine disinfection (GI, >1.6 to >3.7 log_10_; GIII, 1.4 to >3.2 log_10_) than GII and GIV. After ultraviolet disinfection, infectious FRNAPH GII was detected in all ultraviolet-treated samples, whereas GIII was not detected. GI and GIV were detected in ultraviolet-treated samples collected on 11/27 and 11/13, respectively. The highest inactivation among all infectious FRNAPH genotypes was observed for GIII (>2.4–>3.2 log_10_).

Figure 2B shows the number of hits for each FRNAPH genotype except GI-JS from the sum of the number of hits for each genotype (Supplementary Figure S1) in the secondary effluent, chlorine-treated, and ultraviolet-treated samples collected on 11/13, 11/20, and 11/27. We observed the highest ratio of hits for GII (27–115) among the FRNAPH genotypes from chlorine-treated and ultraviolet-treated samples. In particular, 90 and 115 hits were observed in the 11/27 chlorine-treated sample and the 11/20 ultraviolet-treated sample, respectively. Notably, only GII was detected using IC–RT-PCR–MPN. In contrast, the largest decreases among the infectious FRNAPH genotypes were observed among GI and GIII strains in the chlorine-treated samples (18–87% decrease) and GIII strains in the ultraviolet-treated samples (18–87% decrease). These trends were equivalent to those observed using IC–RT-PCR–MPN. 

## 3. Discussion

The aim of this study was to investigate the diversity of infectious FRNAPH strains in wastewater treatment and disinfection processes using IC–NGS. A total of 32 FRNAPH strains were successfully detected in wastewater samples by IC–NGS (Figure 1). These strains have been first isolated from various sources that include not only sewage and environmental waters but also shellfish and human and animal feces (Table 1). This indicates that multiple FRNAPH strains from various sources accumulate in the influent of WWTPs. DL52 (GI-JS) and HL4-9 (GIII) were predominant, representing more than 30% of FRNAPH strains identified in influent samples from the target WWTP. DL52 and HL4-9 were first isolated from bay water and hog lagoons, respectively (Table 1). HL4-9 has been associated with pig waste. The results of our previous study [6], which investigated the occurrence of FRNAPH genotypes in the same WWTP, suggested that livestock waste was present in the influent. Thus, wastewater related to pig farming may be incorporated into the influent of the target WWTP in this study as well. These results suggest that the identification of FRNAPH strains by IC–NGS could be useful for microbial source tracking; however, further investigation is required to identify infectious FRNAPH strains from more specific sources such as animal feces and abattoir wastewater.

A comparison of the proportions of dominant DL52 and HL4-9 in influent and secondary effluent samples revealed that DL52 decreased to a greater extent than HL4-9 (Figure 1). This indicates that DL52 was more efficiently removed by wastewater treatment than HL4-9. Furthermore, the proportions of most strains of FRNAPH GI and GII were similar or higher in the secondary effluent relative to those in the influent, whereas those of GIII, including HL4-9, were decreased. This suggests that strains of FRNAPH GI and GII are more resistant to wastewater treatment than those of GIII. Previous studies determined by IC–RT-PCR–MPN and RT-qPCR have also shown smaller reductions of GI and GII by wastewater treatment when compared to GIII [6,9]. Thus, the results of this study determined by IC–NGS agree with those of previous research. Conversely, DL52 and DL54, which belong to the same genotype (GI-JS), showed different proportions in influent and secondary effluent samples. The proportions of DL54 in secondary effluent samples collected on 11/20 and 11/27 were similar or higher than those in influent samples, while those of DL52 were significantly lower. This result suggests differences in wastewater treatment efficacy for different strains of the same genotype. However, further investigation is required using RT-qPCR in order to evaluate the removal quantities for each strain.

After chlorine disinfection (Figure 1, Cl), DL20 (GII), DL52 (GI-JS), and T72 (GII) were predominant with >20% for 11/13, 11/20, and 11/27 samples, respectively. In particular, DL52 and T72 were not predominant before chlorination in 11/20 and 11/27 samples (Figure 1, SE), whereas DL20 was predominant in the secondary effluent sample collected on 11/13. This indicates that DL52 and T72 is more resistant to chlorination than other FRNAPH strains. Similarly, whereas DL20 (GII) and HL4-9 (GIII) were predominant before and after ultraviolet disinfection in 11/13 and 11/27 samples (Figure 1, SE and UV), respectively, T72 (GII) was only predominant in the ultraviolet-treated sample collected on 11/20. This also indicates that T72 may be more resistant to ultraviolet disinfection than other FRNAPH strains. Future research should confirm the disinfectant resistance of these strains (DL52 and T72) through experiments using isolates of these strains.

Previous studies of the surface properties and removal of FRNAPH genotypes during water treatment used MS2, GA, Qβ, and SP as representative FRNAPH strains of genotypes GI–GIV [20,21,22]. However, DL20, HL4-9, and FI were more predominant strains of FRNAPH GII, GIII, and GIV in our wastewater samples than GA, Qβ, and SP, respectively. Specifically, SP, which was detected only once (ultraviolet-treated sample collected on 11/13), was rarely found in the wastewater samples. Thus, our results suggest that DL20, HL4-9, and FI are more representative FRNAPH strains of genotypes GII, GIII, and GIV, respectively. 

One of the objectives of this study was to evaluate whether IC–NGS data can effectively reflect the infectivity of FRNAPHs by comparing the detection of infectious FRNAPH genotypes using IC–RT-PCR–MPN and IC–NGS. It should be noted that FRNAPH GII showed a higher concentration and number of hits than FRNAPH genotypes GI, GIII, and GIV when IC–RT-PCR–MPN and IC–NGS were used to analyze chlorine-treated and ultraviolet-treated samples (Figure 2A,B). Further, the largest decreases in the number of hits among all infectious FRNAPH genotypes were observed for GI and GIII strains from secondary effluent to chlorine-treated samples as well as GIII strains from secondary effluent to ultraviolet-treated samples (Figure 2B). These data are consistent with those acquired using IC–RT-PCR–MPN (Figure 2A). These results indicate that the infectivity of FRNAPHs is reflected by the IC–NGS data when infectious FRNAPHs are propagated before performing NGS.

Viral diversity measured by NGS varies among studies because of pre-treatment processes such as nucleic-acid extraction and inherent amplification biases during PCR [37,38]. In the IC–NGS results, specific strains that easily propagated during the pre-propagating procedure prior to NGS were more frequently detected by IC–NGS. If specific strains are easily propagated, the distributions of FRNAPH strains would be similar in all samples. However, the distributions of FRNAPH strains differed between influent, secondary effluent, chlorine-treated, and ultraviolet-treated samples, and between those collected on 11/13, 11/20, and 11/27, except for the influent sample (Figure 1). Thus, the propagating bias may not have affected the results of this study. On the other hand, the distribution of FRNAPH strains may have been affected by the culture conditions (temperature, culture time, using the host strain, etc.) in the pre-propagating procedure of IC–NGS. Thus, further studies are needed to investigate the effect of the culture conditions used for IC–NGS on the distribution of FRNAPH strains.

In conclusion, this study revealed that diverse infectious FRNAPH strains are present in wastewater treatment and disinfection processes by IC–NGS. A total of 32 infectious strains belonging to FRNAPH GI (nine strains), GI-JS (two strains), GII (nine strains), GIII (seven strains), and GIV (five strains) were detected in the wastewater samples from a pilot-scale WWTP. The GI and GII strains were more resistant to wastewater treatment than GIII strains. The IC–NGS results from disinfected samples reflected the infectivity of FRNAPHs. Our results suggest that certain strains exhibit greater resistance to chlorine (DL52, GI-JS; T72, GII) and ultraviolet (T72, GII) disinfection than others from the results of laboratory-scale batch disinfection experiments, using secondary effluent samples. The results of this study will be confirmed by investigating full-scale WWTPs. By identifying disinfectant-resistant strains, it is likely that further research will reveal more effective mechanisms for viral disinfection, thereby reducing viruses at WWTPs for ensuring the hygiene and safety of recreational waters.

## 4. Materials and Methods 

### 4.1. Wastewater Samples

Influent and secondary effluent samples were collected from a pilot-scale WWTP (capacity of 10 m^3^/d), which uses conventional activated sludge treatment with 1700–2100 mg/L of mixed-liquor suspended solids. This WWTP is fed by water from the influent of a full-scale WWTP located in Ibaraki Prefecture, Japan. The influent and secondary effluent samples were collected on November 13, 20, and 27, 2017 (designated 11/13, 11/20, and 11/27, respectively). The characteristics of the influent and secondary effluent samples are summarized in Table 3.

### 4.2. Samples Disinfected Using Chlorine or Ultraviolet Light

Chlorine-treated and ultraviolet-treated samples were collected from laboratory-scale batch disinfection experiments using secondary effluent samples (11/13, 11/20, and 11/27). All batch disinfection experiments employing chlorine or ultraviolet light were performed at room temperature. A free-chlorine stock solution was prepared in Milli-Q water with sodium hypochlorite (Wako, Japan) on the day of use. This stock solution was added to the secondary effluent samples (1000 mL each) at an initial free-chlorine concentration of 2 mg/L for 20 min, after which free-chlorine was neutralized immediately by adding sodium thiosulfate solution (Wako, Osaka, Japan). The residual free-chlorine concentrations were measured every 5 min using the *N,N*-diethyl-p-phenylenediamine method (Hach, Tokyo, Japan) to calculate concentration-time (CT) values. Free-chlorine CT values were the sum of the residual free-chlorine concentration (C) multiplied by the contact time (T) every 5 min for 20 min. The free-chlorine CT values of the chlorine-treated 11/13, 11/20, and 11/27 samples were 4.8, 2.9, and 2.3 mg·min/L, respectively.

A low-pressure ultraviolet lamp (ULO-6DQ; 254 nm; 6 W; Ushio, Tokyo, Japan) was used for laboratory-scale batch ultraviolet disinfection experiments. The ultraviolet lamp was stabilized before conducting experiments by turning it on for at least 40 min before use. The sample (500 mL) was added to sterilized glassware (Ushio) and exposed to ultraviolet light whilst stirring. Ultraviolet fluence was determined using an iodide–iodate actinometer [39,40]. Ultraviolet fluence values of ultraviolet-treated 11/13, 11/20, and 11/27 samples were 22, 30, and 21 mJ/cm^2^, respectively.

### 4.3. IC–NGS Analysis of Infectious FRNAPH Strains

For the NGS analysis, 10 mL of influent samples and 100 mL of secondary effluent, chlorine-treated, and ultraviolet-treated samples were mixed with an equal volume of tryptone-glucose broth (10 g/L tryptone, 1.0 g/L glucose, 8.0 g/L NaCl, 0.3 g/L CaCl_2_, 0.15 g/L MgSO_4_, 20 mg/L kanamycin, and 100 mg/L nalidixic acid). The broth also contained *Salmonella enterica* serovar Typhimurium WG49, which was harvested during the exponential growth period and incubated at 37 °C overnight in order to propagate infectious FRNAPH strains. The propagated sample mixtures (15 mL) were centrifuged (2000 ×*g*, 10 min) and the supernatant was passed through a membrane filter (pore size 0.45 μm, hydrophilic cellulose acetate; Dismic-25cs, Advantec, Dublin, CA, USA) to remove bacteria, including the host strain. The filtrate (12 mL) was purified using a centrifugal filtration device (Amicon Ultra-15; Merck, Billerica, MA, USA) to increase the titres in the FRNAPH strains and remove soluble and low molecular weight components from the filtrate.

After purification, the samples (1 mL) were treated with RNase ONE Ribonuclease (Promega, Madison, WI, USA) (1 unit/50 μL of sample), and the mixture was incubated at 37 °C for 60 min to eliminate free RNA. Following RNase treatment, RNA was extracted using a QIAamp Viral RNA Mini QIAcube Kit (Qiagen, Hilden, Germany) and QIAcube (Qiagen), according to the manufacturer’s protocol, followed by removal of DNA with Baseline-ZERO DNase (Arbrown, Chuo-ku, Japan). Bacterial ribosomal RNA was removed from the DNase-treated samples using a Ribo-Zero Bacteria Kit (Illumina, San Diego, CA, USA) according to the manufacturer’s protocol. Libraries were then prepared using the TruSeq Stranded mRNA Library Prep Kit (Illumina), according to the manufacturer’s protocol, without a purifying mRNA process. The TruSeq Stranded mRNA Library Prep Kit purifies poly(A)-containing mRNAs; however, the mRNAs of FRNAPHs do not contain poly(A) and are therefore excluded from this process. The libraries were subjected to agarose gel electrophoresis using E-Gel EX Agarose Gel (1%; Invitrogen, Carlsbad, CA, USA) with an E-Gel iBase Power System (Invitrogen). The cDNAs (300–600 bp) were then purified using a MonoFas DNA Purification Kit (GL Sciences, Torrance, CA, USA). The qualities and concentrations of purified cDNAs were assessed using an Agilent 2100 Bioanalyzer (Agilent Technologies, Santa Clara, CA, USA) and a Qubit Fluorometer (Invitrogen), respectively. The samples were pooled, and sequencing was performed using a MiSeq paired-end sequencing reaction with the v3 reagent kit (Illumina).

Before assembly of the metagenomic dataset, the quality of the MiSeq paired-end sequences was evaluated using FastQC then quality-trimmed and assembled de novo using Trimmomatic and Trinity, respectively, as implemented in the Galaxy platform (https://galaxy.dna.affrc.go.jp). Contigs >200 bp obtained from the de novo assembly were used as queries to perform a BLASTn version 2.7.1 + search with the NCBI nucleotide collection (nt) to identify significant alignments and the following parameters: A cut-off (e-value) of 10^−3^ and a maximum of one hit per read. The number of hits for FRNAPH strains was defined in order to count the number of FRNAPH strains identified as best hits according to the BLASTn analyses. The MEGAN program (version 6.12.0) was used to assign BLASTn hits for the taxonomy analysis.

### 4.4. IC–RT-PCR–MPN Analysis of Infectious FRNAPH Genotypes

IC–RT-PCR–MPN was performed to quantify the infectious FRNAPH genotypes as previously described [6,34,35]. Infectious FRNAPH genotypes in the samples were primarily propagated overnight at 37 °C by mixing with an equal volume of tryptone-glucose broth containing *S. enterica* WG49 (described above). Genotyping based on RT-PCR was subsequently applied, followed by quantification using the MPN method. The secondary effluent, chlorine-treated, and ultraviolet-treated samples were measured using sample volumes of 100, 10, 1, and 0.1 mL (*n* = 3 each). The detection limit of the secondary effluent, chlorine-treated, and ultraviolet-treated samples was 0.48 log_10_ MPN/L.

## Figures and Tables

**Table 1:** Sources of F-specific RNA bacteriophage (FRNAPH) strains.

FRNAPH Genotype	FRNAPH Strain	Source	Reference
GI	MS2	Sewage	[14,15,16]
	M12	Sewage	[14,15,16]
	DL1	River water	[14,15,16]
	DL2	Bay water	[14,15,16]
	DL13	Oyster	[14,15,16]
	DL16	Bay water	[14,15,16]
	J20	Chicken litter	[14,15,16]
	ST4	Unknown	[14,15,16]
	R17	Sewage	[14,15,16]
	Fr	Dung hill	[14,16]
	JP501	Sewage	[17]
GI-JS	DL52	Bay water	[16]
	DL54	Bay water	[16]
GII	GA	Sewage	[14,15,16,17]
	KU1	Sewage	[14,15,16,17]
	DL10	Mussel	[14,15,16]
	DL20	Clam	[14,15,16]
	T72	Bird	[14,15,16]
	BZ13	Sewage	[17]
	TL2	Sewage	[17]
	JP34	Sewage	[17]
	TH1	Sewage	[17]
GIII	Qβ	Human feces	[14,15,17]
	BR12	Creek water	[14,15]
	BZ1	Sewage	[14,15]
	VK	Sewage	[14,15,17]
	TW18	Sewage	[14,15,17]
	HL4-9	Hog lagoon	[14,15]
	M11	Unknown	[14,15]
	MX1	Sewage	[14,15,17]
GIV	SP	Siamang gibbon	[14,15,17,18]
	FI	Infant	[14,15,17,18]
	BR1	Creek water	[14,15]
	BR8	Creek water	[14,15]
	HB-P22	Bird	[14,15]
	HB-P24	Bird	[14,15]
	NL95	Calf	[14,15]

**Table 2:** Characteristics of influent and secondary effluent samples ^1^.

Date (Month/Day)	Sample ^2^	No. of Total Reads	No. of Contigs	No. of Hits for FRNAPHs (Ratio)	No. of Hits for Bacteria (Ratio), [No. of Hits for *Salmonella enterica* (ratio)] ^3^	No. of not Hit Contigs (Ratio) ^4^
11/13	IN	1,135,519	1218	317 (26%)	584 (48%), [380 (65%)]	200 (16%)
	SE	1,080,326	537	87 (16%)	343 (64%), [261 (76%)]	66 (12%)
	Cl	887,593	611	73 (12%)	476 (78%), [414 (87%)]	30 (5%)
	UV	1,278,120	732	66 (9%)	608 (83%), [548 (90%)]	41 (6%)
11/20	IN	1,070,341	1299	468 (36%)	570 (44%), [459 (81%)]	196 (15%)
	SE	1,019,493	614	160 (26%)	310 (50%), [220 (71%)]	95 (15%)
	Cl	1,033,979	776	91 (12%)	591 (76%), [532 (90%)]	52 (7%)
	UV	1,025,377	821	162 (20%)	577 (70%), [505 (88%)]	36 (4%)
11/27	IN	4,092,357	18,941	551 (3%)	10,471 (55%), [2521 (24%)]	6859 (36%)
	SE	4,900,897	4344	247 (6%)	2537 (58%), [1825 (72%)]	1151 (26%)
	Cl	5,035,503	4370	161 (4%)	3484 (80%), [3217 (92%)]	497 (11%)
	UV	4,102,143	2319	106 (5%)	1655 (71%), [1416 (86%)]	497 (21%)

^1^ The number of hits for each FRNAPH or bacterial genome refers to the number of sequences registering hits for FRNAPH genomes or bacterial reference genomes. The ratio is the percentage of the number of hits relative to the number of total contigs in the sample. ^2^ IN: Influent; SE: Secondary effluent; Cl: Chlorine-treated secondary effluent samples; UV: Ultraviolet-treated secondary effluent samples. ^3^ The ratio shown for *Salmonella enterica* is the number of hits for *Salmonella enterica* relative to the number for all bacteria. ^4^ Not hit contigs refers to the absence of hits for any reference genome.

**Table 3:** Characteristics of influent and secondary effluent samples.

Parameter ^1^	Units	Range	
		IN ^2^	SE ^2^
pH	-	7.1–7.3	6.8–6.9
CODcr	mg/L	120–140	11–14
SS	mg/L	47–78	4.7–6.7
Turbidity	NTU	37–44	1.2–2.8
T-N	mg/L	31–34	15–17
T-P	mg/L	9.4–9.6	4.8–5.2
NH4^+^-N	mg/L	20–24	0.12–0.27

^1^ COD: Chemical oxygen demand; SS: Suspended solids; T-N: Total nitrogen; T-P: Total phosphorus. ^2^ IN: Influent; SE: Secondary effluent.

## Supplementary Materials

The following are available online at https://www.mdpi.com/2076-0817/8/4/217/s1. Figure S1: Numbers of hits for FRNAPH strains representing each genotype in the 12 samples combined with a heat map showing the relative abundance of all FRNAPH strains according to the number of hits in the BLASTn analyses of influent (IN), secondary effluent (SE), chlorine-treated (Cl), and ultraviolet-treated (UV) samples. Blank cells indicate an absence of hits. Green and red cells indicate the lowest and highest values, respectively. Numbers in the heat-map cells indicate the number of hits for samples collected on 11/13, 11/20, and 11/27. Figure S2: Proportions of FRNAPH strains in each genotype combined with a heat map showing the relative abundance of each genotype according to the number of hits in the BLASTn analyses of influent (IN), secondary effluent (SE), chlorine-treated (Cl), and ultraviolet-treated (UV) samples. Blank cells indicate an absence of hits. White and blue (GI), sky blue (GI-JS), red (GII), green (GIII), and purple (GIV) cells indicate the lowest and highest values, respectively. Numbers in the heat-map cells indicate the proportions of FRNAPH strains in each genotype for samples collected on 11/13, 11/20, and 11/27.
