# Supplementary material for: Metagenomic Analysis of Infectious F-Specific RNA Bacteriophage Strains in Wastewater Treatment and Disinfection Processes
Source: Pathogens. 2019 Nov 3;8(4):217. doi: 10.3390/pathogens8040217 (PMC6963950; doi:10.3390/pathogens8040217)
Supplement: Supplementary file 1 [file pathogens-08-00217-s001.pdf]

pathogens

MDPI

Article

Supplementary Material: Metagenomic Analysis of Infectious F-specific RNA Bacteriophage Strains in Wastewater Treatment and Disinfection Processes

Suntae Lee *, Mamoru Suwa and Hiroyuki Shigemura

Innovative Materials and Resources Research Center, Public Works Research Institute, Ibaraki 305-8516, Japan; seontae@pwri.go.jp (S.L.); suwa@pwri.go.jp (M.S.); h-shigemura@pwri.go.jp (H.S.)
* Correspondence: seontae@pwri.go.jp; Tel.: +81-29-879-6765 (S.L.)

FRNAPH genotype	FRNAPH strain	No. positive/ no. tested (%)	No. of hits for FRNAPH strains											
			IN			SE			Cl			UV		
			11/13	11/20	11/27	11/13	11/20	11/27	11/13	11/20	11/27	11/13	11/20	11/27
GI	MS2	12/12 (100)	9	14	16	2	12	13	2	1	7	1	4	6
	DL1	12/12 (100)	12	5	20	2	9	22	2	1	4	2	3	8
	J20	12/12 (100)	4	11	17	5	7	22	2	2	5	1	2	6
	fr	10/12 (83)	2	4	15		3	2	2	6	1	2	5	
	DL16	9/12 (75)	4	6	4	2	6	11	1		5			7
	JP501	5/12 (42)		3	5		1	1				3		
	R17	5/12 (42)		1	2		8	3						1
	ST4	4/12 (33)		2	1		2					1		
	M12	3/12 (25)		2			1			1				
	Sum of the number of hits for GI		31	48	80	11	49	74	9	11	22	10	14	28
GI-JS	DL52	12/12 (100)	46	76	110	6	15	24	5	24	17	4	19	11
	DL54	12/12 (100)	23	25	31	4	9	31	3	5	5	4	4	10
	Sum of the number of hits for GI-JS		69	101	141	10	24	55	8	29	22	8	23	21
GII	DL20	12/12 (100)	13	25	32	16	16	12	18	14	28	17	34	9
	T72	12/12 (100)	3	9	8	5	4	8	10	3	38	1	45	8
	GA	12/12 (100)	10	8	12	7	2	7	9	2	5	4	8	3
	DL10	12/12 (100)	1	7	3	2	3	2	3	4	10	6	14	7
	JP34	9/12 (75)	6	3	5		1	6	4		5	3	2	
	KU1	8/12 (67)	1	3	2	1		2		2	3		7	
	BZ13	6/12 (50)	4				2	2		2		1	2	
	TL2	5/12 (42)			3	1			1		1		2	
	TH1	3/12 (25)			1			1					1	
	Sum of the number of hits for GII		38	55	66	32	28	40	45	27	90	32	115	27
GIII	HL4-9	12/12 (100)	57	81	95	13	22	33	1	2	14	5	5	19
	Qβ	11/12 (92)	38	46	53	3	11	11	1	2	1		1	3
	TW18	11/12 (92)	24	30	28	1	13	9	3	2	1	1		3
	VK	8/12 (67)	23	40	27		2	2			2	2		2
	BR12	7/12 (58)	17	34	31		1	7		1		1		
	BZ1	5/12 (42)	3	12	8		1	4						
	M11	4/12 (33)	1	4	1		3							
	Sum of the number of hits for GIII		163	247	243	17	53	66	5	7	18	9	6	27
GIV	FI	12/12 (100)	12	14	9	15	5	2	4	8	3	2	3	1
	BR1	12/12 (100)	3	1	8	1	1	10	2	8	6	4	1	2
	BR8	5/12 (42)	1	1	2	1				1				
	HB-P22	2/12 (17)		1	2									
	SP	1/12 (8)										1		
	Sum of the number of hits for GIV		16	17	21	17	6	12	6	17	9	7	4	3
Sum of the number of hits for all FRNAPH strains			317	468	551	87	160	247	73	91	161	66	162	106

Supplementary Figure S1. Numbers of hits for FRNAPH strains representing each genotype in the 12 samples combined with a heat map showing the relative abundance of all FRNAPH strains according to the number of hits in the BLASTn analyses of influent (IN), secondary effluent (SE), chlorine-treated (Cl), and ultraviolet-treated (UV) samples. Blank cells indicate an absence of hits. Green and red cells indicate the lowest and highest values, respectively. Numbers in the heat-map cells indicate the number of hits for samples collected on 11/13, 11/20, and 11/27.

FRNAPH genotype	FRNAPH strain	No. positive/ no. tested (%)	Proportions of FRNAPH strains in each genotype (%)											
			IN			SE			Cl			UV		
			11/13	11/20	11/27	11/13	11/20	11/27	11/13	11/20	11/27	11/13	11/20	11/27
GI	MS2	12/12 (100)	29.0	29.2	20.0	18.2	24.5	17.6	22.2	9.1	31.8	10.0	28.6	21.4
	DL1	12/12 (100)	38.7	10.4	25.0	18.2	18.4	29.7	22.2	9.1	18.2	20.0	21.4	28.6
	J20	12/12 (100)	12.9	22.9	21.3	45.5	14.3	29.7	22.2	18.2	22.7	10.0	14.3	21.4
	fr	10/12 (83)	6.5	8.3	18.8		6.1	2.7	22.2	54.5	4.5	20.0	35.7	
	DL16	9/12 (75)	12.9	12.5	5.0	18.2	12.2	14.9	11.1		22.7			25.0
	JP501	5/12 (42)		6.3	6.3		2.0	1.4				30.0		
	R17	5/12 (42)		2.1	2.5		16.3	4.1						3.6
	ST4	4/12 (33)		4.2	1.3		4.1					10.0		
	M12	3/12 (25)		4.2			2.0			9.1				
GI-JS	DL52	12/12 (100)	66.7	75.2	78.0	60.0	62.5	43.6	62.5	82.8	77.3	50.0	82.6	52.4
	DL54	12/12 (100)	33.3	24.8	22.0	40.0	37.5	56.4	37.5	17.2	22.7	50.0	17.4	47.6
GII	DL20	12/12 (100)	34.2	45.5	48.5	50.0	57.1	30.0	40.0	51.9	31.1	53.1	29.6	33.3
	T72	12/12 (100)	7.9	16.4	12.1	15.6	14.3	20.0	22.2	11.1	42.2	3.1	39.1	29.6
	GA	12/12 (100)	26.3	14.5	18.2	21.9	7.1	17.5	20.0	7.4	5.6	12.5	7.0	11.1
	DL10	12/12 (100)	2.6	12.7	4.5	6.3	10.7	5.0	6.7	14.8	11.1	18.8	12.2	25.9
	JP34	9/12 (75)	15.8	5.5	7.6		3.6	15.0	8.9	0.0	5.6	9.4	1.7	
	KU1	8/12 (67)	2.6	5.5	3.0	3.1		5.0		7.4	3.3		6.1	
	BZ13	6/12 (50)	10.5				7.1	5.0		7.4		3.1	1.7	
	TL2	5/12 (42)			4.5	3.1			2.2		1.1		1.7	
	TH1	3/12 (25)			1.5			2.5					0.9	
GIII	HL4-9	12/12 (100)	35.0	32.8	39.1	76.5	41.5	50.0	20.0	28.6	77.8	55.6	83.3	70.4
	Qβ	11/12 (92)	23.3	18.6	21.8	17.6	20.8	16.7	20.0	28.6	5.6		16.7	11.1
	TW18	11/12 (92)	14.7	12.1	11.5	5.9	24.5	13.6	60.0	28.6	5.6	11.1		11.1
	VK	8/12 (67)	14.1	16.2	11.1		3.8	3.0			11.1	22.2		7.4
	BR12	7/12 (58)	10.4	13.8	12.8		1.9	10.6		14.3		11.1		
	BZ1	5/12 (42)	1.8	4.9	3.3		1.9	6.1						
	M11	4/12 (33)	0.6	1.6	0.4		5.7							
GIV	FI	12/12 (100)	75.0	82.4	42.9	88.2	83.3	16.7	66.7	47.1	33.3	28.6	75.0	33.3
	BR1	12/12 (100)	18.8	5.9	38.1	5.9	16.7	83.3	33.3	47.1	66.7	57.1	25.0	66.7
	BR8	5/12 (42)	6.3	5.9	9.5	5.9				5.9				
	HB-P22	2/12 (17)		5.9	9.5									
	SP	1/12 (8)										14.3		

Supplementary Figure S2. Proportions of FRNAPH strains in each genotype combined with a heat map showing the relative abundance of each genotype according to the number of hits in the BLASTn analyses of influent (IN), secondary effluent (SE), chlorine-treated (Cl), and ultraviolet-treated (UV) samples. Blank cells indicate an absence of hits. White and blue (GI), sky blue (GI-JS), red (GII), green (GIII), and purple (GIV) cells indicate the lowest and highest values, respectively. Numbers in the heat-map cells indicate the proportions of FRNAPH strains in each genotype for samples collected on 11/13, 11/20, and 11/27.
